# Ready-to-eat cereal is an affordable breakfast option associated with better nutrient intake and diet quality in the US population

**DOI:** 10.3389/fnut.2022.1088080

**Published:** 2023-01-09

**Authors:** Yong Zhu, Neha Jain, James Normington, Norton Holschuh, Lisa M. Sanders

**Affiliations:** ^1^Bell Institute of Health and Nutrition, General Mills, Golden Valley, MN, United States; ^2^Statistics and Data Science, General Mills, Mississauga, ON, Canada; ^3^Statistics and Data Science, General Mills, Golden Valley, MN, United States; ^4^Cornerstone Nutrition, LLC, Battle Creek, MI, United States

**Keywords:** ready-to-eat cereal, breakfast, meal cost, dietary intake, nutrition affordability

## Abstract

**Background:**

Results from observational studies have reported ready-to-eat cereal (RTEC) consumers have higher dietary quality and nutrient intake compared to consumers of non-RTEC breakfasts or those who do not eat breakfast. Yet, there have been few investigations on the relationship of RTEC to meal costs at breakfast and across the day, which may be one reason some consumers choose to not consume breakfast.

**Objective:**

The objective of this study is to evaluate the contribution of RTEC consumed at breakfast to nutrient intake and adequacy, diet quality and meal costs in a nationally representative sample of children and adults in the US.

**Methods:**

Dietary data from 2,259 children (2–18 years) and 4,776 adults (≥19 years) in the National Health and Nutrition Examination Survey (NHANES) 2017–2018 were evaluated to compare nutrient intake, adequacy, dietary quality, and food costs in RTEC breakfast consumers, non-RTEC breakfast consumers and those who did not consume breakfast.

**Results:**

RTEC breakfast consumers made up 28% of children and 12% of adults. Children and adults consuming RTEC for breakfast had higher intakes of carbohydrate, dietary fiber, calcium, magnesium, iron, zinc, phosphorus, potassium, B vitamins, vitamins A and D, whole grains, and total dairy compared to consumers of non-RTEC breakfast or no breakfast. There were no differences by breakfast status for sodium, saturated fat, or added sugar, except adults consuming RTEC had lower added sugar intake compared to those who did not consume breakfast. RTEC breakfast consumers were also more likely to meet estimated average requirements (EAR) for intake of several nutrients and had overall higher dietary quality. For children, breakfast meal costs were less for RTEC breakfast compared to non-RTEC breakfast, but total daily meal costs were similar for consumers of RTEC and non-RTEC breakfasts.

**Conclusion:**

RTEC breakfasts may contribute to greater nutrient intake and diet quality in children and adults in the US without increasing total daily meal costs.

## 1. Introduction

Breakfast is considered an important component of a healthy diet and eating a healthy breakfast is often recommended by health professionals and governments around the world (1). Consumption of breakfast has been associated with better dietary quality, a healthy body weight, better cognitive performance, and better cardiovascular health compared to breakfast skippers (1–8). Yet a decline in breakfast consumption has been noted in many countries around the globe, including the US (3, 9).

Ready-to-eat cereal is a frequent breakfast choice in the US, particularly with children and adolescents (6, 7, 10). Evidence from observational studies report higher diet quality and better nutrient intake among RTEC breakfast consumers compared to non-RTEC breakfasts and breakfast skippers, including higher intakes of fiber, B vitamins, and iron, as well as lower intakes of saturated fat (1, 7, 8, 10, 11). RTEC intake has also been associated with greater intake of typically under-consumed food groups, such as dairy and whole grains (1, 6).

RTEC is also recognized as a convenient, acceptable, and affordable food and is integral to many federal nutrition assistance programs. Financial constraints in low-income households may limit the ability of families to purchase the quantity and quality of food necessary to meet their nutrient needs. Additionally, individuals in low-income households may be more likely to skip a meal, such as breakfast, to save money (12). The global survey of Health Behaviour in School-age Children reported adolescents from less affluent households across numerous countries are less likely to consume breakfast daily than adolescents in more affluent households (3). As an affordable, nutrient-fortified option, RTEC may be able help children and adults from low-income households meet their nutrient needs with less strain on finances as some other breakfast options (13).

While some studies have reported positive associations of RTEC consumption on nutrient intake and diet quality in food insecure and low-income households (10, 14, 15), no studies have examined the relationship of RTEC on breakfast meal and daily food costs. The purpose of this study was to evaluate the contribution of RTEC consumed at breakfast to nutrient intake and adequacy, diet quality, and meal costs in a nationally representative sample of children and adults in the US. It was hypothesized that RTEC intake would be associated with better diet quality and higher intake of vitamins, minerals, fiber and whole grains, compared to non-RTEC breakfasts and no breakfast. Additionally, it was hypothesized that breakfast meals consisting of RTEC would cost less than other breakfasts and result in lower total daily food costs.

## 2. Materials and methods

### 2.1. Participants

The present study used data from the National Health and Nutrition Examination Survey (NHANES) 2017–2018 (16). The NHANES is a nationally representative survey that examines nutrition and health status of non-institutionalized Americans; since 1999, the data is released every 2 years and includes data from interviews, medical examination, and 24-h dietary recalls conducted by trained interviewers (17). In NHANES 2017–2018, there were 7,483 participants who provided day-1 24-h dietary records as reliable dietary recall as determined by NHANES. Excluding 362 infants and toddlers younger than 2 years old and 86 pregnant or lactating women, the present study involves 2,259 children aged 2–18 years and 4,776 adults aged 19 years or older.

### 2.2. Breakfast status

Breakfast was defined as “breakfast” or “desayuno” eating occasion from the day-1 24-h dietary recall data. Participants were classified as breakfast skippers if there was no such eating occasion reported in their dietary recalls or if total caloric intake from breakfast was less than 50 kcals (8). Those who consumed breakfast were classified as ready-to-eat cereal (RTEC) breakfast or non-RTEC breakfast depending on whether any RTEC food was reported in their breakfast meal and the total caloric intake from breakfast was at least 50 kcal; whereas RTEC was identified by food codes from RTEC food categories in What We Eat in America (18).

### 2.3. Dietary outcomes

The NHANES 2017–2018 dietary data were linked with USDA Food and Nutrient Database for Dietary Studies (FNDDS) 2017–2018 (19) and Food Patterns Equivalents Database (FPED) 2017–2018 (20); Energy and nutrient intake were obtained from day-1 dietary recalls. Food groups including added sugar, whole grains, refined grains, and total dairy products were obtained from day-1 FPED data. Nutrition adequacy was assessed as percentages below Estimated Average Requirement (EAR) calculated using the National Cancer Institute Usual Intake method (21). Contribution of breakfast to daily energy or nutrient intake was estimated as energy or nutrient intake from breakfast divided by total daily energy or nutrient intake. Diet quality was assessed as the Healthy Eating Index 2015 (HEI-2015), which is a measurement on compliance with 2015–2020 Dietary Guidelines for Americans (22).

### 2.4. Food cost

Similar to the approach used to derive a food cost database for NHANES 2013–2016 in previous studies (13, 23), food cost was based on the most recent, publicly available NHANES food price database (2001–2004) developed by the USDA Center for Nutrition Policy and Promotion (24). Food codes in NHANES 2017–2018 were matched with food codes in NHANES 2001–2004; any new food codes were hand-matched to the most closely matching food code; after matching, the food price was adjusted for inflation using Consumer Price Index from Bureau of Labor Statistics (25) to derive food cost in NHANES 2017–2018. Breakfast meal cost and total day meal cost were calculated as sum of price for individual foods consumed, and were presented as direct cost, as well as cost per 2,000 kcal.

### 2.5. Covariates

Similar to previous studies (6, 7), age, gender (male, female), race/ethnicity (Non-Hispanic White, Non-Hispanic Black, Mexican American, Other Hispanic, Other Race), and ratio of family income to poverty (≤1.85, 1.86–3.49, ≥3.50) were obtained from demographic data and used as the list of covariates in analysis of energy, nutrient, and food group intake, as well as analysis of the HEI-2015 score. In addition, energy intake was added as another covariate in analysis of nutrient and food group intake.

### 2.6. Statistical analysis

SAS 9.4 (SAS Institute, Cary, NC, USA) was used for statistical analysis. SAS survey procedures were applied with 2-year sample weight to account for the multi-stage survey design in NHANES. Differences in dietary outcomes and meal costs by breakfast status were compared using survey linear regression, adjusted for covariates as described previously, followed by Bonferroni-corrected *P* values from three pair-wise comparisons among different breakfast status (*P* < 0.05/3 = 0.0167). Results were presented as weighted percentage or least square means with standard errors. Data from children and adults were analyzed separately.

## 3. Results

### 3.1. Demographic characteristics of participants

Among children, 18.6% were breakfast skippers, 28.1% were RTEC breakfast consumers, and 53.3% were non-RTEC breakfast consumers. The percentages were 23.1, 12.2, and 64.6% in adults, respectively. Table 1 includes age, gender, race/ethnicity, and ratio of family income to poverty. Older children and younger adults were more likely to be breakfast skippers. There was no difference in breakfast status between boys and girls, however, adult men were more likely to be breakfast skippers than women. Race/ethnicity was not associated with breakfast status in children; however, there was a higher percentage of non-Hispanic black adults who were breakfast skippers. No difference was found in the distribution of ratio of family income to poverty by breakfast status in children, however, there was a higher percentage of adults from lower income families who skipped breakfast.

**TABLE 1:** Demographic characteristics of US children and adults by breakfast status, National Health and Nutrition Examination Survey, 2017–2018.

	Children 2–18 years				Adults ≥ 19 years			
	**RTEC breakfast**	**Non-RTEC breakfast**	**No breakfast**	***P*-value**	**RTEC breakfast**	**Non-RTEC breakfast**	**No breakfast**	***P*-value**
	**(*n* = 612)**	**(*n* = 1,190)**	**(*n* = 457)**		**(*n* = 516)**	**(*n* = 3,119)**	**(*n* = 1,141)**	
Age, mean ± SE, years	9.0 ± 0.3	9.8 ± 0.3	12.8 ± 0.2	<0.0001	52.8 ± 1.3	49.4 ± 0.6	42.4 ± 0.8	<0.0001
Gender, *n* (%)								
Male	303 (27.8%)	603 (55.2%)	210 (17.0%)	0.174	271 (12.3%)	1,481 (61.4%)	613 (26.2%)	0.001
Female	309 (28.5%)	587 (51.2%)	247 (20.3%)		245 (12.1%)	1,638 (67.7%)	528 (20.1%)	
Race/Ethnicity, n (%)								
Non-Hispanic White	233 (29.9%)	387 (53.4%)	136 (16.8%)	0.061	272 (14.7%)	1,086 (64.7%)	350 (20.5%)	<0.0001
Non-Hispanic Black	138 (25.4%)	252 (50.7%)	119 (23.9%)		102 (10.0%)	670 (56.1%)	356 (33.8%)	
Mexican American	105 (32.1%)	195 (48.5%)	87 (19.4%)		42 (5.9%)	433 (67.0%)	156 (27.1%)	
Other Hispanic	34 (16.3%)	81 (60.7%)	34 (22.9%)		32 (8.8%)	329 (72.1%)	81 (19.1%)	
Other race	102 (24.8%)	275 (57.6%)	81 (17.6%)		68 (7.5%)	601 (66.6%)	198 (25.9%)	
Ratio of family income to poverty, n (%)								
≤1.85	328 (31.0%)	533 (47.5%)	254 (21.5%)	0.060	196 (11.2%)	1,161 (59.7%)	498 (29.1%)	0.002
1.86–3.49	121 (24.5%)	268 (55.6%)	100 (19.9%)		124 (15.0%)	701 (63.3%)	250 (21.8%)	
≥3.50	96 (27.3%)	287 (59.5%)	57 (13.3%)		150 (12.0%)	885 (68.4%)	249 (19.6%)	

### 3.2. Energy, nutrients, and food group intake

Table 2 presents daily intake of energy, nutrients, and food groups by breakfast status in children and adults. In both children and adults, those who did not consume breakfast had a significantly lower energy intake, however, there was no difference in energy intake between RTEC breakfast and non-RTEC breakfast. Significantly higher intake of carbohydrate, dietary fiber, total sugar, calcium, magnesium, iron, zinc, phosphorus, potassium, vitamin A, thiamin, riboflavin, niacin, vitamin B6, folate, vitamin B12, vitamin D, whole grains, total dairy was found in children who consumed RTEC breakfast; they also had lower intake of total fat than children who did not consume breakfast and lower intake of total fat and refined grains than children who consumed non-RTEC breakfast (all *P* < 0.0167). No difference in intake of protein, sodium, vitamin C, vitamin E, saturated fat, and added sugar was found by breakfast status in children. The results were generally similar in adults, except there were no differences in total sugar and fat intake and lower added sugar intake in adults consuming RTEC breakfasts compared to those who did not consume breakfast. Additionally, non-RTEC adult, breakfast eaters had higher protein, fiber, total fat, saturated fat, calcium, iron, potassium, vitamin A, thiamin, riboflavin and vitamin C intake and lower total and added sugar intake than breakfast skippers (all *P* < 0.0167).

**TABLE 2:** Adjusted daily intake of energy, nutrients and food groups in US children and adults by breakfast status, National Health and Nutrition Examination Survey, 2017–2018.

	Children 2–18 years				Adults ≥ 19 years			
	**RTEC breakfast**	**Non-RTEC breakfast**	**No breakfast**	***P*-value**	**RTEC Breakfast**	**Non-RTEC Breakfast**	**No Breakfast**	***P*-value**
	**(*n* = 612)**	**(*n* = 1190)**	**(*n* = 457)**		**(*n* = 516)**	**(*n* = 3,119)**	**(*n* = 1,141)**	
Energy (kcal)	1,922.3 ± 39.0a	1,952.5 ± 29.0a	1,580.8 ± 52.4b	0.0001	2,215.8 ± 70.3a	2,213.9 ± 20.3a	1,874.9 ± 25.1b	<0.0001
Carbohydrate (g)	255.6 ± 2.2a	239.3 ± 2.2b	235.2 ± 2.6b	<0.0001	275.3 ± 4.8a	244.7 ± 1.4b	250.6 ± 3.6b	<0.0001
Dietary fiber (g)	15.8 ± 0.7a	14.2 ± 0.3b	13.6 ± 0.4b	0.0127	21.9 ± 0.6a	17.9 ± 0.4b	16.0 ± 0.5c	<0.0001
Total sugars (g)	114.7 ± 2.1a	105.2 ± 1.7b	106.7 ± 2.5b	0.0056	117.6 ± 3.5a	101.6 ± 1.4b	111.4 ± 2.6a	0.0003
Total fat (g)	69.1 ± 0.7b	74.8 ± 0.6a	77.1 ± 1.2a	<0.0001	78.4 ± 2.1b	85.3 ± 0.5a	82.8 ± 0.8b	0.0058
Saturated fat (g)	24.3 ± 0.5a	25.4 ± 0.3a	26.0 ± 0.7a	0.0112	25.5 ± 0.6ab	27.2 ± 0.2a	26.1 ± 0.3b	0.0045
Protein (g)	63.8 ± 1.1a	65.5 ± 1.2a	63.7 ± 1.3a	0.3381	79.7 ± 1.2ab	83.4 ± 0.8a	76.8 ± 1.4b	0.0018
Calcium (mg)	1,087.8 ± 27.5a	930.7 ± 18.2b	858.7 ± 22.9b	<0.0001	1111.6 ± 35.3a	936.3 ± 12.4b	812.2 ± 20.9c	<0.0001
Magnesium (mg)	248.9 ± 4.6a	231 ± 3.4b	211.9 ± 4.7c	0.0001	345.2 ± 4.8a	310.1 ± 3.8b	278.7 ± 7.6c	0.0002
Iron (mg)	18.0 ± 0.3a	11.6 ± 0.2b	11.5 ± 0.3b	<0.0001	21.9 ± 0.5a	13.5 ± 0.1b	12.5 ± 0.2c	<0.0001
Zinc (mg)	11.7 ± 0.3a	8.2 ± 0.2b	8.6 ± 0.3b	<0.0001	13.4 ± 0.3a	10.7 ± 0.1b	10.0 ± 0.3b	<0.0001
Phosphorus (mg)	1284.2 ± 20.6a	1225.4 ± 16.7a	1137.3 ± 18.4b	<0.0001	1478.8 ± 22.4a	1380.9 ± 12.5b	1257.0 ± 18.5c	<0.0001
Potassium (mg)	2220.9 ± 43.7a	2094.4 ± 35.2b	1975.1 ± 35.2b	0.0003	2912.0 ± 59.1a	2649.7 ± 29.5b	2431.9 ± 45.5c	0.0001
Sodium (mg)	2,755.5 ± 60.3a	2905.5 ± 31.4a	2,931.6 ± 43.5a	0.0673	3,269.3 ± 48.2b	3,497.1 ± 36a	3,471.4 ± 54.3ab	<0.0001
Vitamin A, RAE (μg)	767.7 ± 19.4a	526.2 ± 14.9b	483.0 ± 21.4b	<0.0001	928.5 ± 40.6a	637.7 ± 22.4b	489.0 ± 24.4c	<0.0001
Thiamin (mg)	1.9 ± 0.1a	1.4 ± 0.1b	1.3 ± 0.1b	<0.0001	2.0 ± 0.1a	1.6 ± 0.0b	1.4 ± 0.0c	<0.0001
Riboflavin (mg)	2.2 ± 0.1a	1.6 ± 0.1b	1.6 ± 0.1b	<0.0001	2.4 ± 0.1a	2.0 ± 0.1b	1.7 ± 0.1c	<0.0001
Niacin (mg)	24.5 ± 0.5a	19.3 ± 0.4b	19.8 ± 0.8b	<0.0001	28.8 ± 0.6a	25.1 ± 0.4b	24.3 ± 0.8b	0.0002
Vitamin B6 (mg)	2.2 ± 0.1a	1.5 ± 0.1b	1.5 ± 0.1b	<0.0001	2.7 ± 0.1a	2.1 ± 0.1b	1.9 ± 0.1b	<0.0001
Folate, DFE (μg)	726.5 ± 15.8a	395.3 ± 7.2b	401.5 ± 17.8b	<0.0001	843.5 ± 21.8a	465.5 ± 8b	435.0 ± 12.7b	<0.0001
Vitamin B12 (μg)	6.1 ± 0.2a	3.4 ± 0.1b	3.7 ± 0.2b	<0.0001	6.7 ± 0.3a	4.4 ± 0.1b	4.2 ± 0.2b	<0.0001
Vitamin C (mg)	81.9 ± 7.1a	78.0 ± 4.7a	70.8 ± 4.9a	0.3752	86.7 ± 4.8ab	87.1 ± 2.6a	74.3 ± 2.9b	0.0014
Vitamin D ((μg)	6.4 ± 0.2a	4.0 ± 0.1b	3.5 ± 0.2b	<0.0001	7.1 ± 0.3a	4.2 ± 0.1b	3.4 ± 0.4b	<0.0001
Vitamin E (mg)	8.0 ± 0.5a	7.4 ± 0.2a	7.6 ± 0.3a	0.4383	9.4 ± 0.4a	9.4 ± 0.2a	8.7 ± 0.4a	0.3836
Added sugar (tsp eq.)	15.9 ± 0.5a	15.0 ± 0.5a	16.8 ± 0.7a	0.0562	16.0 ± 0.8b	15.1 ± 0.4b	19.1 ± 0.6a	<0.0001
Whole grains (oz eq.)	1.2 ± 0.1a	0.8 ± 0.1b	0.5 ± 0.1c	<0.0001	1.4 ± 0.2a	0.8 ± 0.1b	0.4 ± 0.1c	<0.0001
Refined grains (oz eq.)	5.6 ± 0.1b	6.2 ± 0.1a	5.8 ± 0.2ab	0.0064	5.4 ± 0.2a	6.0 ± 0.1a	5.9 ± 0.2a	0.1017
Total dairy (cup eq.)	2.1 ± 0.1a	1.7 ± 0.1b	1.5 ± 0.1b	0.0000	1.8 ± 0.1a	1.2 ± 0.1b	1.1 ± 0.1b	0.0000

Data presented are least square mean with standard error, adjusted for age, gender, race/ethnicity, and ratio of family income to poverty; nutrient and food groups data were also adjusted for energy intake. Different letters in the same row within the data panel for children or adults indicate a significant difference from Bonferroni-corrected post hoc comparison (*P* < 0.0167). RAE, retinal activity equivalents; DFE, dietary folate equivalents.

### 3.3. Nutrient adequacy

Table 3 includes percent below EAR for key vitamins and minerals in children and adults by breakfast status. Both children and adults who skipped breakfast were generally more likely to have intake below EAR; whereas those who consumed RTEC breakfast were less likely to have intake below EAR.

**TABLE 3:** Percent below estimated average requirement (EAR) in US children and adults by breakfast status, National Health and Nutrition Examination Survey, 2017–2018.

	Children 2–18 years			Adults ≥ 19 years		
	**RTEC breakfast**	**Non-RTEC breakfast**	**No breakfast**	**RTEC breakfast**	**Non-RTEC breakfast**	**No breakfast**
	**(*n* = 612)**	**(*n* = 1,190)**	**(*n* = 457)**	**(*n* = 516)**	**(*n* = 3,119)**	**(*n* = 1,141)**
Thiamin	1 ± 0%	3 ± 1%	13 ± 2%	2 ± 0%	7 ± 1%	21 ± 2%
Riboflavin	0 ± 0%	2 ± 0%	10 ± 2%	2 ± 1%	5 ± 1%	15 ± 1%
Niacin	1 ± 0%	2 ± 0%	6 ± 1%	1 ± 0%	3 ± 1%	7 ± 1%
Vitamin B6	1 ± 0%	4 ± 1%	17 ± 2%	6 ± 1%	14 ± 1%	27 ± 2%
Folate, DFE	1 ± 0%	6 ± 1%	30 ± 4%	3 ± 1%	13 ± 1%	36 ± 2%
Vitamin B12	0 ± 0%	2 ± 1%	12 ± 2%	2 ± 1%	7 ± 1%	18 ± 1%
Zinc	7 ± 1%	18 ± 2%	43 ± 3%	10 ± 1%	22 ± 2%	43 ± 2%
Vitamin A, RAE	10 ± 1%	27 ± 2%	60 ± 2%	16 ± 2%	41 ± 2%	72 ± 2%
Vitamin C	10 ± 2%	21 ± 2%	42 ± 3%	37 ± 2%	50 ± 2%	64 ± 2%
Iron	0 ± 0%	2 ± 1%	13 ± 3%	0 ± 0%	2 ± 0%	10 ± 1%
Calcium	34 ± 3%	51 ± 2%	80 ± 2%	23 ± 3%	41 ± 2%	59 ± 1%
Vitamin D	89 ± 2%	98 ± 1%	100 ± 0%	85 ± 2%	97 ± 1%	100 ± 0%

RAE, retinal activity equivalents; DFE, dietary folate equivalents.

### 3.4. Diet quality

Children and adults who consumed RTEC breakfast had significantly higher HEI-2015 total score than non-RTEC breakfast eaters or breakfast skippers (all *P* < 0.0167, Table 4). For sub-scores, RTEC breakfast eaters had higher scores for whole grains, dairy, refined grains, and lower scores for fatty acids in both children and adults compared to non-RTEC breakfast eaters and/or breakfast skippers (all *P* < 0.0167). For adults who had RTEC breakfast, they also had higher scores for sodium than non-RTEC breakfast; whereas those who had non-RTEC breakfast had higher scores for added sugar than breakfast skippers (all *P* < 0.0167).

**TABLE 4:** Healthy Eating Index (HEI)-2015 total score and sub-scores in US children and adults by breakfast status, National Health and Nutrition Examination Survey, 2017–2018.

		Children 2–18 years				Adults ≥ 19 years			
	**Maximum score**	**RTEC breakfast**	**Non-RTEC breakfast**	**No breakfast**	***P*-value**	**RTEC breakfast**	**Non-RTEC Breakfast**	**No Breakfast**	***P*-value**
		**(*n* = 612)**	**(*n* = 1,190)**	**(*n* = 457)**		**(*n* = 516)**	**(*n* = 3119)**	**(*n* = 1141)**	
Total vegetables	5	2.0 ± 0.1a	2.1 ± 0.1a	2.2 ± 0.1a	0.2354	3.2 ± 0.1a	3.1 ± 0.1a	3.1 ± 0.1a	0.5326
Greens and beans	5	1.1 ± 0.2a	1.0 ± 0.1a	1.0 ± 0.1a	0.7717	1.9 ± 0.2ab	1.9 ± 0.1a	1.4 ± 0.1b	0.0006
Total fruits	5	5.0 ± 0.0a	5.0 ± 0.0a	5.0 ± 0.0a	0.9999	5.0 ± 0.0a	5.0 ± 0.0a	5.0 ± 0.0a	0.3854
Whole fruit	5	2.4 ± 0.2a	2.4 ± 0.1a	2.1 ± 0.2a	0.1603	2.9 ± 0.1a	2.3 ± 0.1b	1.7 ± 0.1c	<0.0001
Whole grains	10	4.1 ± 0.3a	2.6 ± 0.1b	2.0 ± 0.2b	<0.0001	4.2 ± 0.2a	2.4 ± 0.1b	1.5 ± 0.1c	<0.0001
Dairy	10	7.4 ± 0.2a	6.0 ± 0.1b	5.4 ± 0.3b	<0.0001	6.2 ± 0.2a	4.2 ± 0.1b	3.7 ± 0.2c	<0.0001
Total protein food	5	3.3 ± 0.1a	3.7 ± 0.1a	3.7 ± 0.1a	0.0455	4.0 ± 0.1b	4.3 ± 0.0a	4.1 ± 0.1ab	0.0108
Seafood and plant proteins	5	1.7 ± 0.2a	1.8 ± 0.1a	1.5 ± 0.2a	0.0548	2.8 ± 0.1a	2.6 ± 0.1a	2.1 ± 0.1b	0.0001
Fatty acids	10	3.4 ± 0.2b	4.3 ± 0.2a	4.5 ± 0.3a	0.0001	4.6 ± 0.3b	5.3 ± 0.1ab	5.6 ± 0.1a	0.0055
Sodium	10	5.8 ± 0.2a	5.3 ± 0.2a	5.1 ± 0.2a	0.1540	5.1 ± 0.2a	4.4 ± 0.1b	4.5 ± 0.2ab	0.0089
Refined grains	10	5.3 ± 0.2a	4.6 ± 01b	5.0 ± 0.3ab	0.0030	6.7 ± 0.3a	5.8 ± 0.2ab	5.6 ± 0.2b	0.0101
Saturated fat	10	5.4 ± 0.2a	5.1 ± 0.2a	5.1 ± 0.3a	0.2177	6.2 ± 0.2a	5.7 ± 0.1a	6.2 ± 0.2a	0.0256
Added sugar	10	6.4 ± 0.2a	6.7 ± 0.2a	6.2 ± 0.2a	0.0825	7.0 ± 0.2ab	7.2 ± 0.1a	6.4 ± 0.1b	<0.0001
Total HEI-2015	100	53.4 ± 0.8a	50.7 ± 0.4b	48.7 ± 1.2b	0.0009	59.7 ± 0.9a	54.2 ± 0.5b	50.8 ± 0.6c	<0.0001

Data presented are least square mean with standard error, adjusted for age, gender, race/ethnicity, and ratio of family income to poverty. Different letters in the same row within the data panel for children or adults indicate a significant difference from Bonferroni-corrected post hoc comparison (*P* < 0.0167).

### 3.5. Contribution of breakfast to daily energy and nutrient intake

**FIGURE 1:**
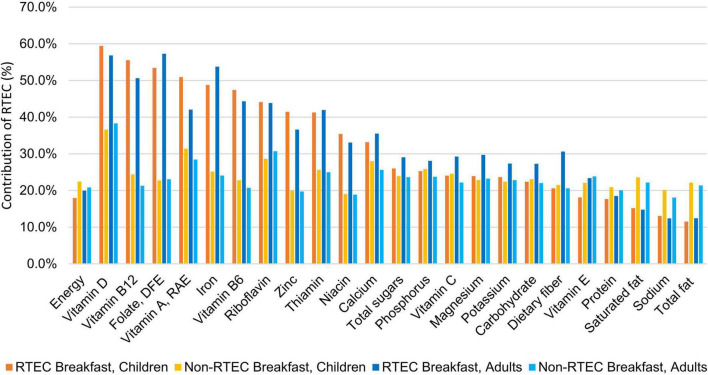
Contribution of ready-to-eat cereal (RTEC) breakfast and non-ready-to-eat cereal breakfast to daily intake of energy and nutrients in breakfast eaters in the US, National Health and Nutrition Examination Survey, 2017–2018.

Figure 1 presents the contribution of RTEC breakfast and non-RTEC breakfast to energy and nutrient intake in children and adults. Approximately 20% of energy intake was from breakfast among breakfast consumers in children and adults. RTEC breakfast however, contributed a larger percent of key vitamins and minerals such as vitamin D, vitamin B12, folate, vitamin A, iron, vitamin B6, riboflavin, zinc, and niacin. By contrast, non-RTEC breakfast contributed a larger percent of saturated fat, sodium, and total fat.

### 3.6. Meal cost

**FIGURE 2:**
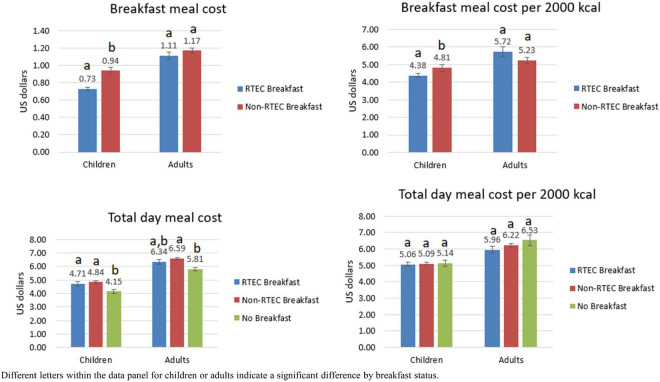
Breakfast meal cost and total day meal cost by breakfast status, National Health and Nutrition Examination Survey, 2017–2018.

Results on meal cost are presented in Figure 2. The cost of breakfast was lower for RTEC breakfast than non-RTEC breakfast in children (*P* < 0.0001), but the costs were not significantly different in adults (*P* = 0.187); the results were similar when breakfast meal cost was standardized to a 2,000 kcal diet. Total day meal cost in children was lower in breakfast skippers than both RTEC breakfast eaters and non-RTEC breakfast eaters, and the total day meal cost in adults was also lower in breakfast skippers than non-RTEC breakfast eaters (all *P* < 0.0167). However, these differences become non-significant when the cost was standardized to a 2,000 kcal diet.

## 4. Discussion

The results of this study demonstrate adults and children in the US consuming an RTEC breakfast have greater intake of several commonly under-consumed nutrients, as well as higher intakes of whole grains and dairy compared to consumers of non-RTEC breakfasts or breakfast skippers. RTEC breakfasts also contributed substantially to the intake of several of these nutrients, including vitamin D, iron, and B vitamins. RTEC consumers were more likely to meet nutrient requirements and have better overall diet quality compared to non-RTEC consumers and breakfast skippers, which is consistent with previous findings in the US and other countries (6, 7, 10, 26–28). For children only, breakfast meal costs were lower for RTEC breakfast compared to non-RTEC breakfast, and total daily meal costs were similar for consumers of RTEC breakfast, non-RTEC breakfast and no breakfast, when standardized by calories.

More than one quarter of children surveyed reported consuming RTEC for breakfast, yet only 12.2% of adults reported consuming RTEC, suggesting RTEC consumption declines with age in favor of non-RTEC breakfasts or skipping breakfast. Other studies have reported similar findings (7, 11). Interestingly, breakfast status in children was not significantly impacted by sex, race/ethnicity, or family income to poverty ratio, but there were significant impacts in adults. Consistent with other research (29), men were more likely to skip breakfast than women, although these results were somewhat surprising given that breakfast skipping among adolescents has been reported primarily in females (29–31). Additionally, adults from low-income families were more likely to skip breakfast than higher income families, which deserves further investigation. A study of women from low sociodemographic areas in Australia reported breakfast skipping was associated with other poor lifestyle habits, such as smoking and lower consumption of fruits and vegetables, a lack of nutrition knowledge, less family support for healthy food choices, and not prioritizing health to take care of their family (32), while other studies have reported cost and time constraints are common reasons for meal skipping in general (29). Further research is needed to identify specific barriers in low-income populations in the US and evaluate opportunities to overcome these barriers to encourage breakfast consumption as part of a healthy diet.

Both adult and child RTEC consumers had greater intakes of most vitamins and minerals and were more likely to meet the recommended intake for these nutrients compared to breakfast skippers and non-RTEC breakfasts, in agreement with previous studies (6, 7, 33, 34). These greater intakes were most likely attributable to RTEC which contributed between 30 and 60% of daily nutrients, such as vitamin D, vitamin B12, folate, vitamin A, iron, vitamin B6, riboflavin, zinc, and niacin, while contributing ≤20% of daily energy. In adults, RTEC contributed almost one third of daily dietary fiber intake which is frequently under consumed and linked to reduced risk of cardiovascular disease, type 2 diabetes and some cancers (35). While the daily energy contribution of non-RTEC breakfasts were similar to RTEC breakfasts, they delivered higher amounts of total fat, saturated fat and sodium compared to RTEC breakfasts, suggesting RTEC can be nutrient dense choice for breakfast to help consumers meet nutritional needs without excess calories or undesirable nutrients.

RTEC consumers also had better diet quality compared to non-RTEC consumers and those who consumed no breakfast, consistent with previous research (6, 7, 10, 36). While the current results cannot evaluate causation, the more favorable sub-group scores for whole grain and dairy, which are key components of an RTEC breakfast, suggest RTEC may play an important role in improving overall diet quality. Other studies have reported RTEC breakfasts provide more than half of daily whole grain intake and more than 60% of daily dairy intake in adults and children (7, 37). Additionally, sub-scores for added sugar or saturated fat did not differ from non-RTEC breakfasts or no breakfast conditions, further reinforcing the potential contribution of RTEC breakfasts to diet quality.

Despite concerns about the sugar content of breakfast cereals, these data suggest RTEC consumers did not have higher added sugar intake than consumers of non-RTEC breakfasts or those who did not consume breakfast. In fact, in adults, daily added sugar intake was greater in those who did not consume breakfast compared to RTEC and non-RTEC breakfasts. Some studies have reported skipping meals is associated with increased snacking, which may contribute to higher added sugar intake (30, 31). These findings are consistent with previous research that has reported RTEC to not be a major contributor to added sugar intake in adults and children (6, 7). In fact, national survey data in the US and Australia reported breakfast cereals contribute approximately 2.5–3% of added sugars in the diet, with values slightly higher in the UK (4.8–7.9%) (38–41).

In addition to higher nutrient intakes and dietary quality, RTEC breakfasts were also more affordable for children compared to non-RTEC breakfasts, even when standardized for calories. This suggests RTEC is a cost-effective way to deliver nutrients and food groups to achieve better diet quality in children. Other studies have also suggested RTEC as a low-cost option to improve nutrient intakes and diet quality in low income and food insecure children (10, 14, 15). Interestingly, costs for RTEC breakfasts and non-RTEC breakfasts were similar in the adult population. This may be due to the costs of other meal components common to RTEC and non-RTEC breakfasts in adults, such as coffee and fruit.

It was not surprising that those who do not eat breakfast have lower direct costs for daily food, but when considered on a calorie basis, there is no apparent cost savings for those who choose to not consume breakfast. This is critical information for consumers, particularly those with lower incomes, that may be choosing to skip breakfast as a potential cost saving measure. Not only was there lack of cost savings, but potential further disadvantage to nutrient intake and diet quality with skipping the breakfast meal.

Strengths of this study include the use of a large, representative sample of children and adults in the US population. Additionally, dietary intake was measured using a validated 24-h recall with an automated multiple-pass method that captures detailed food information, including branded items, allowing for more precise nutrient determinations. However, there are some limitations, such as the cross-sectional and observational nature of the findings that limit the ability to determine causality. Furthermore, the use of 24-h recalls may not reflect the usual dietary intake of the subject or the frequency of consumption of RTEC. This study also only assessed RTEC intake at breakfast and not other eating occasions. While RTEC is predominantly consumed at breakfast, consuming RTEC at other times of the day can have an equally important impact on nutrient intake and dietary quality as has been described in other studies (7, 14, 38, 42, 43). Finally, the food price database for NHANES 2001–2004, adjusted for inflation over time, may over or underestimate the true cost of meals in NHANES 2017–2018.

In conclusion, this study demonstrates RTEC breakfast consumption was associated with improved nutrient intake and dietary quality in adults and children. The cost of breakfast was less for children consuming RTEC compared to non-RTEC breakfasts, but there was no difference in total daily meal costs for adults and children consuming RTEC compared to non-RTEC breakfasts. These findings will be critical for communications with low-income and food insecure households that may choose to skip breakfast as a cost-saving measure yet may not realize these savings and may also miss out critical nutrients and food groups that an RTEC breakfast can provide. Food policy makers and federal nutrition feeding programs may also benefit from this information as they seek low-cost, nutrient dense food options to improve food and nutrition security for children and adults in the US.

## Data availability statement

Publicly available datasets were analyzed in this study. This data can be found here: https://wwwn.cdc.gov/nchs/nhanes/Default.aspx.

## Author contributions

YZ designed research. NJ, JN, and NH analyzed the data and performed statistical analysis. YZ and LS wrote the article. All authors interpreted the results and have read and approved the final content.
